# Mathematical Modeling of *Salmonella* Inactivation During Apple Drying and Pre-Drying Heating in Closed Environments

**DOI:** 10.3390/foods13233877

**Published:** 2024-11-30

**Authors:** Ren Yang, Shuang Zhang, Juming Tang

**Affiliations:** 1 Department of Agricultural and Biosystems Engineering, South Dakota State University, P.O. Box 2100, SRPA-136, Brookings, SD 57007, USA; 2 Department of Biological Systems Engineering, Washington State University, P.O. Box 646120, Pullman, WA 99164, USA; shuang.zhang@wsu.edu; 3 Department of Industrial & Systems Engineering, University of Washington, P.O. Box 352650, Seattle, WA 98195, USA

**Keywords:** apple, drying, *Salmonella*, thermal, inactivation, modeling, humidity

## Abstract

Drying is one of the most effective preservation methods for extending the shelf-life of perishable foods. The microbial safety of low-moisture food products had not been recognized as a concern until outbreaks reported over the past decade in products contaminated with bacterial pathogens, in particular *Salmonella*. There is now an urgent need to understand the influence of process conditions on the thermal inactivation of pathogens in various drying operations. This study aimed to develop a predictive model for *Salmonella* inactivation in diced apples during hot air drying and in high-humidity heating in closed environments. Fresh-cut apple cubes (6 mm) inoculated with a cocktail of *Salmonella enterica* strains (Enteritidis PT30, Montevideo 488275, and Agona 447967) were placed in a customized box inside an oven for three different treatments: (1) open-box drying at oven temperature 90 °C (Drying-90); (2) close-box pre-drying heating at 90 °C (PD heating-90); and (3) close-box pre-drying heating at 70 °C (PD heating-70). Air temperature, relative humidity (RH), and sample temperatures were monitored, and *Salmonella* survival was measured at multiple time intervals. After 10 min, the air RH reached 66% in PD heating-90 and 74% in PD heating-70, versus 30% in Drying-90. A 5-log reduction in *Salmonella* was achieved in 8.5 min in PD heating-90, and 14 min in PD heating-70, compared to 28.7 min in Drying-90. A mathematical model using sample surface RH and sample temperature profiles accurately predicted *Salmonella* inactivation across all treatments (RMSE = 0.92 log CFU/g, R^2^ = 0.86), with thermal death parameters comparable to isothermal studies. This study underscores the role of humidity in enhancing microbial reduction during drying and proposes high-humidity pre-drying heating as an effective control step. The developed model shows promise for real-time prediction of microbial inactivation in complex drying environments with dynamic temperature and humidity conditions.

## 1. Introduction

Hot air drying is a popular unit operation in the commercial production of a wide range of low-moisture ready-to-eat food products, including dehydrated fruits, vegetables, herbs, and protein sources such as meat, eggs, plant-based proteins, and dairy products. Drying removes moisture from food, reducing the water activity of the product to inhibit enzymatic activities and the growth of microorganisms, i.e., bacteria, yeasts and molds [1,2]. While hot air drying had traditionally been assumed effective in reducing microbial contamination, certain bacterial pathogens, particularly *Salmonella*, demonstrate high thermal resistance in low-moisture environments. It was reported that the thermal resistance of *Salmonella* increases exponentially with the decrease in relative humidity. For example, the *D*-value (time required to cause 1 log reduction) for *Salmonella* Enteritidis PT 30 at 80 °C was 159 min at 18% relative humidity (RH), which is over 80 times higher than 1.8 min at 72% RH [3]. Thus, *Salmonella* may survive some drying processes, rendering the products unsafe for direct consumption. *Salmonella* has been implicated in nationwide outbreaks and recalls in United States across a wide range of fresh and dehydrated products, including onions, basil, and dried fruits and spices [4,5,6,7,8]. Research suggests that *Salmonella*’s ability to attach to or internalize in fruits and vegetables significantly contributes to the contamination of susceptible products [8]. The average cost of a food recall is estimated at USD 10 million, with additional losses stemming from brand damage and decreased sales [9]. The recalls are more costly for long-shelf-life low-moisture foods, leading to an average capitalization loss of over USD 400 million per event [10]. The persistence of heat-resistant pathogens in dehydrated food products represents a growing concern with serious public health and economic implications for the U.S. food system.

Traditional drying processes, designed primarily to maximize efficiency and preserve product quality, frequently fall short in achieving the necessary inactivation levels for bacterial pathogens. For example, spray drying has been shown to achieve less than a 3-log reduction in *Salmonella* even with air temperatures of 180 °C [11]. Hot air drying of fresh cabbage leaves at 60 °C only achieved a less than 2-log reduction in *Salmonella* over the complete drying operation [12]. Other studies indicate that *Salmonella* can survive certain drying conditions due to this increased resistance in low-moisture, desiccated environments [4,13,14,15]. Several recent studies have provided convincing evidence that the desiccation of vegetative bacterial cells enhances their thermal resistance, making them more difficult to inactivate [14,16,17,18].

To comply with the Food Safety Modernization Act (FSMA) and minimize contamination risks, the food industry has adopted two main strategies: (1) adding a validated pasteurization step, such as steam treatment and propylene oxide (PPO) fumigation [19]; or (2) validating that the existing (legacy) processes are able to deliver the adequate level of microbial inactivation. Although adding pasteurization steps improves safety, it increases production costs and resource demands. Additionally, for fresh ingredients like fruits and vegetables, there have been very few established and effective pasteurization methods. Validating individual food drying operations are costly. Currently, food processors often use surrogate inoculation challenge studies to validate lethality under specific conditions [20,21]. However, these studies cannot provide real-time predictions of microbial inactivation or indicate conditions under which the process may fail, especially when accounting for product variability, raw material inconsistencies, process fluctuations, and an increasing prevalence of pathogens linked to climate change [22,23,24]. Thus, a reliable mathematical model that can predict microbial inactivation based on process parameters is critical for ensuring the antimicrobial efficacy of drying processes.

Recent studies have shown that microbial inactivation in low-moisture environments is closely influenced by product temperature and the humidity immediate around the products [3,15,18,25,26]. While microbial inactivation modeling is well established for high-moisture products in thermal processes, there has been limited exploration of microbial reduction prediction in complex drying processes where temperature and humidity are dynamic. This study addresses this gap by assessing the impact of temperature and humidity on microbial lethality to provide safer, more efficient drying practices. The goal was to develop a predictive model for *Salmonella* inactivation in diced apples under various drying and pre-drying heating conditions.

## 2. Materials and Methods

### 2.1. Sample Preparation

Fresh whole snack-size Gala apples were purchased from local stores in Pullman, WA, USA, stored in a refrigerator at 4 °C, and used within two weeks of purchase. Apples with consistent quality and texture were peeled, cored, and diced into 6 × 6 × 6 mm cubes using a commercial vegetable dicer (Dynamic CL003, Dynamic International Ltd., Montreal, QC, Canada). The initial moisture content of fresh apples was measured as 86.5% (w.b.), using the AOAC 934.06 method [27].

### 2.2. Microbial Inoculation

A cocktail of three *Salmonella enterica* strains—*S. Enteritidis* PT30 (ATCC BAA-1045), *S. Montevideo* (488275), and *S. Agona* (447967)—was prepared using a lawn-based method to achieve a target concentration of *Salmonella* at approx. 9.5 CFU/g apple [28]. Each strain was cultured in a separate tryptic soy broth (TSB, Bacto™, Sparks, MD, USA) with 0.6% yeast extract (TSBYE) through two consecutive incubations (24 h each at 37 °C). Cultures were then plated on tryptic soy agar (TSA, Difco™, Sparks, MD, USA) with yeast extract (TSAYE) for 24 h at 37 °C. The bacterial lawn was scraped into buffered peptone water (BPW), centrifuged (3000× *g*, 15 min), and resuspended in BPW to form a three-strain cocktail. The cocktail was mist-sprayed onto 150 g of apple cubes inside a sterile glass mason jar, with thorough shaking after each spray to ensure even distribution.

### 2.3. Thermal Treatment Conditions

**Figure 1:**
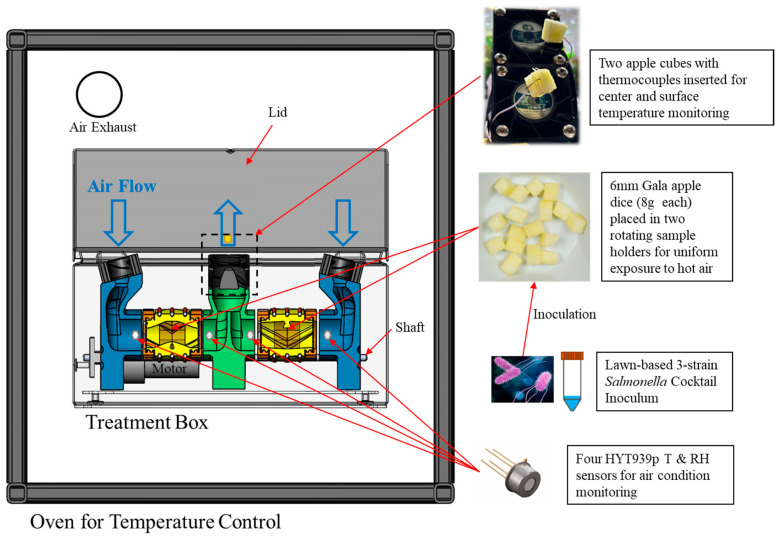
Schematic diagram of the equipment for thermal treatment (drying and pre-drying heating) and physical property monitoring (figure adapted from [25]).

A sample treatment box, designed in a previous study [25] to enhance sample heating uniformity and air condition monitoring, was used in this research (Figure 1). Inoculated apple cubes (8 g each) were loaded into two drum-shaped sample holders with mesh screens on both sides. These holders were placed inside the preheated treatment box within a convection oven (HCP50, Memmert, Schwabach, Germany) preset at 70 °C or 90 °C, the lower and higher ends of typical temperature range for apple drying. The oven door was sealed, while the box lid was open (for hot air drying) or closed (for high-humidity heating).

The tests were conducted under one of three different conditions: (1) oven air at 90 °C, box lid was open for open-air drying, (Drying-90); (2) 90 °C, box lid was closed (PD heating-90); and (3) 70 °C, box lid was closed (PD heating-70). The open-box setup allowed air circulation between the oven and treatment box, with an exhaust vent open to release humid air. The close-box setup retained moisture inside, creating a high-humidity environment while still allowing heat conduction. A motor rotated the sample holders at 37 rpm to enhance the sample surface to hot air exposure, and four internal fans (11,000 rpm, 12 Volt, 0.16 Amps) forced hot air through the holders, enhancing heat and moisture transfer as well as heating uniformity.

Samples were taken out of the treatment box after 5–7 different time periods, appropriate time intervals and overall test durations were selected for each treatment condition (i.e., Drying-90: 0, 3, 6, 12, 18, 24, 36 min; PD heating-90: 0, 2, 3, 4, 6, 8, 9, 10 min; PD heating-70: 0, 4, 8, 12, 16 min) based on preliminary tests to allow observations of more than 5-log *Salmonella* reductions.

### 2.4. Data Collection

At the end of each treatment, the oven and the treatment box (if closed) were opened to allow ambient air to flush into the sample holders to cool the samples. Then each sample (initially 8 g) was transferred into a 50-mL centrifugal tube with 32 mL BPW and vortexed for 5 min to release *Salmonella*. Serial dilutions were prepared using BPW solutions and then plated on TSAYE plates supplemented with 0.05% (*w*/*v*) ferric ammonium citrate (Sigma-Aldrich, St. Louis, MO, USA), and 0.03% (*w*/*v*) sodium thiosulfate (Mallinckrodt Baker, Phillipsburg, NJ, USA) for colony counting after 24 h incubation at 37 °C. Colonies with dark spots were counted, and the colony forming unit (CFU) per gram of apple cubes (initial mass) was calculated for each sample. Each condition was tested at least three times.

During each treatment, air temperature and RH were monitored using four humidity and temperature sensor modules (HYT939P, Innovative Sensor Technology, Ebnat-Kappel, Switzerland), positioned near the air inlet and outlet of each sample holder (Figure 1). Sample temperatures were monitored by high-accuracy K-type thermocouples (0.08 mm diameter): one located at the geometric center of an apple cube (uninoculated) and the other tied to the surface of a second apple cube with a cotton thread about 5 mm away from the tip. The sensor modules and thermocouples were connected to a customized data logger (configured with Arduino Mega 2560 Rev3) for communication, calibration, and data recording (with an SD card). Prior to the tests, calibration was performed in a high-temperature environmental chamber (HCP50, Memmert) at temperatures of 70, 80, and 90 °C, and RH levels of 10, 30, 50, and 70%. The temperature and relative humidity were recorded every second over the duration of each treatment (20 min for PD heating-70, 10 min for PD heating-90, and 45 min for Drying-90).

### 2.5. Statistical and Modeling Analysis

The first-order kinetics was used for *Salmonella* inactivation to describe thermal inactivation of *Salmonella* under isothermal and iso-relative humidity conditions [3,25,29,30]. For drying operations in which product temperature and relative humidity changed with time, the following relationship (adapted from [31]) was used to calculate accumulated lethality:

(1) $$log\frac{N_{0}}{N}=\frac{1}{D_{ref}}\cdot \int_{0}^{t}10^{\left(\frac{T-T_{ref}}{Z_{T}}\cdot \frac{RH-{RH}_{ref}}{Z_{RH}}\right)}\cdot dt$$

where *N*_0_ and *N* are the initial and final microbial population (CFU/g sample, initial mass), *T* and *RH* are current temperature and relative humidity, *T_ref_* and *RH_ref_* are reference temperature and humidity, *D_ref_* is the *D*-value at the reference condition, *Z_T_* and *Z_RH_* represent changes in temperature and relative humidity, respectively, required for a one-log reduction in *D*-value, and *t* is the thermal treatment time.

Considering that the drying process is dynamic and nonequilibrium, sample surface *RH* was used in Equation (1), it was calculated as:

(2) $$sample\;surface\;RH=\frac{air\;AH}{surface\;saturation\;AH}\times 100\%$$

where *AH*, or absolute humidity (the mass of water vapor per unit volume in g/m^3^), was calculated based on the temperature and *RH* of air near the sample holders’ outlets:

(3) $$Air\;AH=\frac{RH}{100\%}\cdot {AH}_{s.T_{air}}$$

and surface saturation *AH* corresponded to the absolute humidity at sample surface temperature. Saturated *AH* at a given temperature (*T*) was calculated using the Ideal Gas Law:

(4) $${AH}_{s.T}=\frac{M\cdot P_{s.T}}{R(T+273.15)}$$

where *M* is the molar mass of water (18.01528 g/mol), *R* is the gas constant (8.3145 m^3^PaK^−1^mol^−1^), and *P*_s*.T*_ is the saturated water vapor pressure at temperature *T* (°C), calculated using Buck’s equation: [32]:

(5) $$P_{s.T}=611.21\;exp\left(\left(18.678-\frac{T}{234.5}\right)\left(\frac{T}{T+257.14}\right)\right)$$

Model fitting for Equation (1) was performed using the Solver function of Microsoft Excel (Version 2406) to minimize the sum of squared residuals. Model fit quality was evaluated using root mean squared error (RMSE) and the coefficient of determination (R^2^) using the Equations (6) and (7), respectively:

(6) $$RMSE=\sqrt{\frac{\sum_{i=1}^{n}{\left[{log\left(\frac{N}{N_{0}}\right)}_{data,i}-{log\left(\frac{N}{N_{0}}\right)}_{model,i}\right]}^{2}}{n-p}}$$

where ${log\left(N/N_{0}\right)}_{data,i}$, and ${log\left(N/N_{0}\right)}_{model,i}$ are the measured and modeled log reduction in survival population of *Salmonella*, *n* is the number of data points, and *p* is the degree of freedom.

(7) $$R^{2}=1-\frac{\sum_{i=1}^{n}\left[{log\left(\frac{N}{N_{0}}\right)}_{data,i}-{log\left(\frac{N}{N_{0}}\right)}_{model,i}\right]}{\sum_{i=1}^{n}\left[{log\left(\frac{N}{N_{0}}\right)}_{data,i}-\bar{log\left(\frac{N}{N_{0}}\right)}\right]}$$

where $\bar{log\left(N/N_{0}\right)}$ is the mean of observed log reductions.

*Salmonella* log reductions at different time points were compared within each treatment group using one-way ANOVA (MATLAB R2024b), with significance set at *p* < 0.05.

## 3. Results and Discussion

### 3.1. Humidity and Temperature Profiles

**Figure 2:**
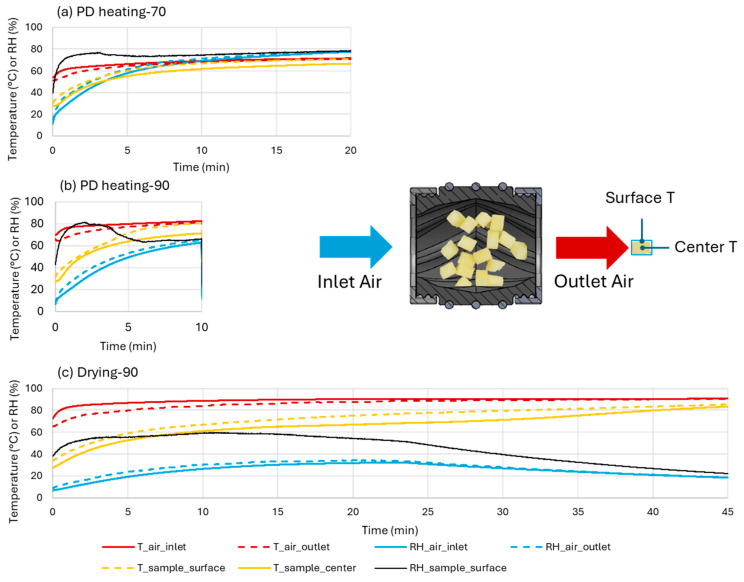
The calculated (sample surface RH) and measured (all the others) profiles of physical properties of the air and apple cubes in drying at 90 °C (**c**) and pre-drying heating treatments at 70 °C (**a**) and 90 °C (**b**).

Figure 2 shows the measured sample surface and internal temperatures, as well as air temperature and relative humidity (RH) at the inlet and outlet of the sample holder over the course of a test under each of the three studied conditions. In the conventional drying operation (Drying-90) (Figure 2c), sample surface and center temperatures increased rapidly in the beginning until 10 min when the sample center and surface temperatures reached 61.0 and 66.9 °C, respectively. Then they gradually reached 68.8 and 77.6 °C at 25 min, and ended at 83.4 and 85.3 °C. The hot air temperature at the inlet started at 72.6 °C upon sample addition, then stabilized at 88.7 °C within 10 min. The air inlet and outlet RH reached a plateau at 30.2 and 33.1% by 15 min and gradually declined to 18.4 and 18.6% by 45 min. The calculated sample surface RH rose from 38% to 55% within the first 4 min and remained between 55 and 60% until 18.5 min.

In the PD heating-90 treatment (Figure 2b), the sample center temperature reached 71.4 °C within 10 min, approximately 9.6 °C higher than in Drying-90. The surface temperature reached 80.5 °C, 13.6 °C higher than in the Drying-90 condition, with near-equilibrium achieved between the sample surface and outlet air temperatures by 10 min (∆T < 0.1 °C). However, the close-box treatment reduced heat transfer, with the hot air temperature reaching only 80.5 °C, 8.2 °C lower than in Drying-90. *RH* in the closed box rose above 60% within 10 min, significantly higher than that (<30%) observed in Drying-90. Sample surface *RH* spiked to 80% within 0.8 min, gradually decreased to 64 ± 2% at 6 min, and remained stable until the end of 10 min. The increased humidity within the closed treatment box limited moisture evaporation from the sample, accelerating temperature equilibration between the sample and hot air.

In the PD heating-70 treatment (Figure 2a), sample center and surface temperatures reached 61.6 °C and 66.9 °C within 10 min, respectively. The air inlet temperature reached 69 °C by 10 min, only 11.5 °C lower than in PD heating-90 despite the oven setting being 20 °C lower. Air *RH* rose to 67%, higher than in PD heating-90, likely due to the reduced moisture capacity of the cooler air. Sample surface *RH* reached 75% within 3 min and maintained a narrow range between 73 and 78.5% throughout the 20 min. The higher sample surface *RH* in PD heating-70 contributed to a reduced surface temperature, which in turn lowered the surface absolute humidity upon saturation.

Overall, closing the treatment box significantly increased air humidity and sample temperature by retaining the evaporated water vapor within the sample box. This raised sample surface *RH*, particularly during the initial four minutes of the PD heating-90 treatment and throughout PD heating-70. The elevated air humidity increased the external water vapor pressure, thereby reducing moisture evaporation from the apple cubes. This reduction in evaporation allowed the sample temperature to rise more rapidly. In commercial operations, similar effects can be achieved by limiting air exhaust, and this effect can be quantified by monitoring air temperature and relative humidity.

### 3.2. Salmonella Survival During Treatment

Table 1 presents the survival populations of inoculated *Salmonella* (three-strain cocktail) and the corresponding log reductions (mean ± standard deviation). The initial inoculation levels were consistent across samples, averaging 9.5–9.6 log (CFU/g sample, initial mass). In the PD heating-70 treatment, a microbial reduction in 3.3 ± 0.8 log was achieved within 12 min. Similarly, PD heating-90 resulted in a 3.5 ± 1.3 log reduction in just 6 min—half the time required in PD heating-70. In contrast, Drying-90 showed only a 1.0 ± 0.3 log reduction at the initial 12 min of drying. 24 min was needed to cause a comparable 3.2 log reduction, which was twice the time of PD heating-70 and four times that of PD heating-90. At the first significant log reduction point for each treatment, sample temperatures were above 63 °C. Despite similar product temperature profiles in the first 12 min of Drying-90 and PD heating-70, the significantly lower sample surface RH in Drying-90 likely contributed to reduced microbial inactivation.

### 3.3. Microbial Reduction Modeling

**Figure 3:**
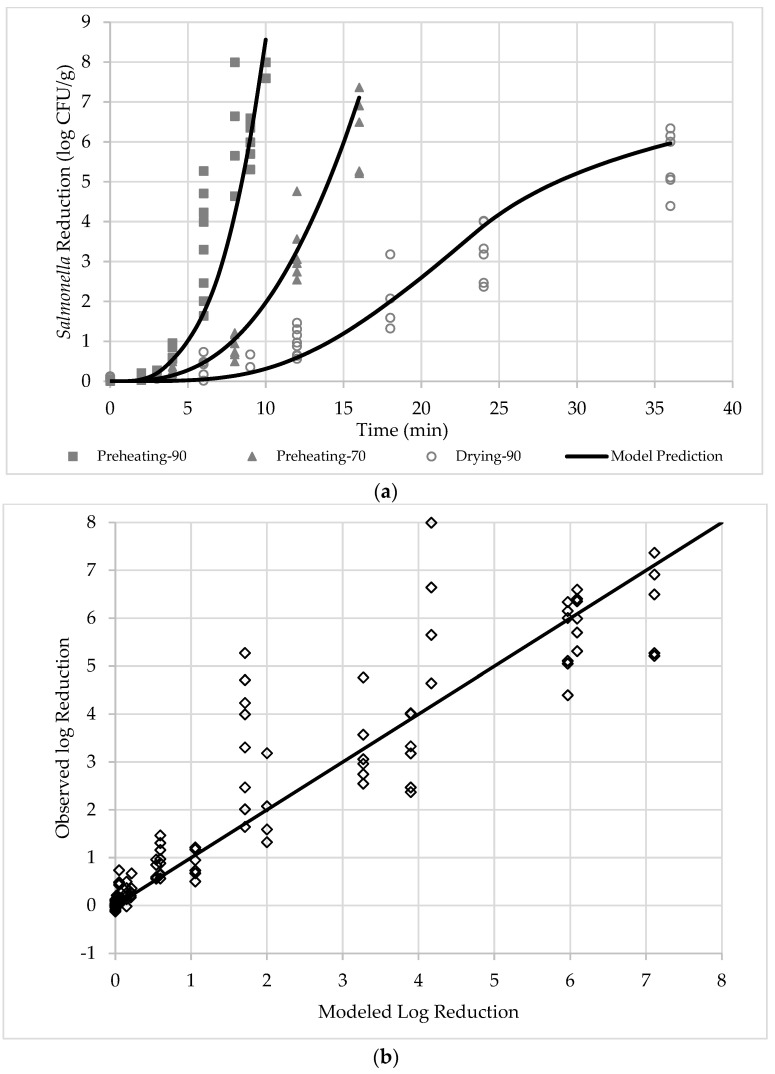
Figure illustration of the observed and modeled reduction (line) in *Salmonella* population in apples at three different treatments: (**a**) reduction plotted vs treatment time; (**b**) observed vs predicted plot (n = 1).

The observed reductions in *Salmonella* for the three treatments are shown in Figure 3, alongside modeled reductions for visual comparison. Modeled parameters for Equation (1), RMSE, and R^2^ are provided in Table 2. With an RMSE of 0.92 and R^2^ of 0.86, the model accurately predicted *Salmonella* reduction for all the treatments. Although some observed reductions deviated by up to 4 logs from the model, all instances of overestimation were within 1.9 logs of the prediction line, suggesting that the model tends to be conservative. Increased uncertainty in observed reductions over time may be attributed to variations in physical conditions across batches. Monitoring sample and air properties for each microbial test could further improve model accuracy in future studies.

The modeled curves in Figure 3a indicate an accelerated microbial inactivation in the pre-drying heating treatments. In Drying-90, microbial reduction followed an S-shaped curve, with the highest inactivation rate at 23.7 min—after which there was a rapid drop in sample surface *RH*, from 51% to 22%. This suggests that microbial inactivation is most effective when sample temperatures exceeded 60 °C and surface *RH* remained above 50%. In the later stage of drying, reduced surface *RH* increases bacterial thermal resistance, slowing inactivation [18].

According to the model, the time required to achieve a 5-log reduction in *Salmonella* was 8.5 min in PD heating-90, 14.0 min in PD heating-70, and 28.7 min in Drying-90. The moisture retention in the close-box pre-drying heating treatments significantly accelerated microbial reduction, likely due to (1) elevated air *RH*, which also increased sample surface *RH*, and (2) reduced moisture evaporation, which allowed for faster sample temperature increases. The model indicates that total microbial inactivation during conventional drying can be reliably predicted by measuring sample surface temperature and air conditions within the drying chamber. Further studies are needed to assess how sample size, initial product temperature, and wider range of drying air temperature would influence microbial reduction in drying operations.

### 3.4. Comparing Modeled Thermal Death Parameters with Literature Data

Table 3, Table 4 and Table 5 compare the modeled *D*_ref_ (70 °C, 80% RH), *Z*_T_, and *Z*_RH_ values with values from the literature. The *D*_ref_ value for *Salmonella* (three-strain cocktail) in this study was 0.60 min, aligning with values for *Salmonella* in grape juice concentrate and apple/orange juice, and closely matching a recent hot ascorbic acid treatment study on apples (*D*_ref_ = 0.63 min) [33]. This suggests that *D*_ref_ values derived from nonlinear treatments and isothermal studies are comparable [34]. *D*_ref_ values for low-moisture foods were significantly higher (7.5 and 11.5 folds greater), likely due to the lower acidity in fruit juices, which reduces the thermal resistance of *Salmonella*. Similar effects have been observed in studies showing that blanching or acid immersion increases microbial inactivation during drying [4,12,35,36].

Table 4 presents *Z*_T_ values, which range from 6.7 to 20.6 °C; the value decreased with increasing *RH*. The fitted *Z*_T_ value from this study (12.6 °C) aligns with values observed at *RH* levels between 25 and 53%, which is reasonable given the broad sample surface *RH* ranges in the treatments (Figure 2). Model accuracy for future applications could be improved by accounting for *Z*_T_ temperature dependence. Table 5 shows *Z*_RH_ values ranging from 21 to 44% across the studied food matrices and processing conditions, with the value from this study closely matching the data from a *Salmonella* inactivation study on humidity-controlled thermal treatment of black peppercorns [25]. No clear correlation between the treatment conditions and the *Z*_RH_ values of *Salmonella* was observed. The discrepancies among *Z*_RH_ values reported from different studies may be attributed to different measurement methods or estimation for RH, or the influence of different food matrices. Further research is needed to better understand the factors that determine *Z*_RH_ value.

Overall, the nonlinear model provided reliable predictions for the thermal death parameters of *Salmonella*. Given the substantial workload required to obtain *Z*_T_ and *Z*_RH_ values under isothermal and constant humidity conditions, nonlinear modeling offers a viable alternative.

## 4. Conclusions

This study provides valuable insights into the role of product surface humidity and temperature in *Salmonella* inactivation during apple drying and pre-drying heating under controlled environments. By comparing traditional drying (Drying-90) with two high-humidity pre-drying treatments (PD heating-90 and PD heating-70), we demonstrate that keeping water vapor in a closed environment during product heating significantly enhances microbial inactivation. The close-box pre-drying treatments achieved faster reductions in *Salmonella* populations, with a 5-log reduction obtained in only 8.5 min at 90 °C and 14.0 min at 70 °C, compared to 28.7 min in conventional drying at 90 °C. These results highlight the positive impact of elevated humidity in reducing bacterial thermal resistance, particularly during the initial heating stages, enabling faster and more efficient microbial inactivation. The use of high-humidity pre-drying presents a promising intervention step for achieving more rapidly and efficiently microbial control, potentially reducing operational time and energy consumption in industrial drying processes. This approach may also enable the production of better product quality by reducing the need for elevated drying temperatures or extended drying times in low-humidity environments.

A nonlinear mathematical model was successfully developed in this study to predict *Salmonella* reduction using surface temperature and relative humidity profiles. By calculating sample surface humidity, this model provided accurate predictions across all the treatments, with thermal death parameters consistent with values reported in the literature on isothermal studies.

This study was limited to diced apples, and further research is needed to validate these findings across a broader range of food matrices and drying conditions. Expanding this approach to diverse food products will be essential for establishing humidity-controlled pre-drying heating as a versatile microbial control strategy in the food industry. Future work should also investigate variations in sample temperature and surface RH across the depth of the product bed, which will be important for adapting this model to commercial drying operations. Additionally, advancements in real-time monitoring technologies and further refinement of the model to account for the humidity impact on *Z*_T_ value could optimize food safety interventions in industrial drying operations.

## Figures and Tables

**Table 1:** Observed and modeled reduction in *Salmonella* population in apples at different experimental conditions (n = 2–6).

Treatment	Time (min)	Log *N* (log CFU/g)	[Log *N*_0_ − Log *N*] * (log CFU/g)	Modeled Reduction (log CFU/g)
PD heating-70	0	9.57 ± 0.15	0.00 ± 0.01 ^a^	0.00
	4	9.33 ± 0.28	0.24 ± 0.18 ^a^	0.15
	8	8.70 ± 0.37	0.87 ± 0.29 ^a^	1.03
	12	6.30 ± 0.87	3.27 ± 0.81 ^b^	3.15
	16	3.21 ± 1.04	6.36 ± 0.91 ^c^	6.77
PD heating-90	0	9.56 ± 0.07	0.00 ± 0.04 ^A^	0.00
	2	9.37 ± 0.07	0.13 ± 0.08 ^A^	0.02
	3	9.38 ± 0.05	0.23 ± 0.05 ^A^	0.20
	4	8.76 ± 0.19	0.74 ± 0.19 ^A^	0.52
	6	6.10 ± 1.33	3.46 ± 1.32 ^B^	1.66
	8	3.27 ± 1.44	6.23 ± 1.43 ^C^	4.03
	9	3.52 ± 0.50	6.06 ± 0.49 ^C^	5.86
	10	1.70 ± 0.28	7.80 ± 0.28 ^C^	8.20
Drying-90	0	9.51 ± 0.15	0.00 ± 0.10 ^a^	0.00
	3	9.43 ± 0.05	0.10 ± 0.05 ^ab^	0.00
	6	9.16 ± 0.26	0.36 ± 0.25 ^ab^	0.05
	12	8.56 ± 0.27	0.96 ± 0.33 ^b^	0.61
	18	7.41 ± 0.75	2.04 ± 0.82 ^c^	2.04
	24	6.28 ± 0.59	3.23 ± 0.71 ^d^	3.97
	36	4.05 ± 0.76	5.51 ± 0.77 ^e^	6.16

* Differences among grouping letters within the same treatment indicate significant differences.

**Table 2:** The model and fitted parameters for the prediction of *Salmonella* reduction in diced apple cubes from drying treatment under three different treatments.

Model	$log\frac{N_{0}}{N}=\frac{1}{D_{ref}}\cdot \int_{0}^{t}10^{\left(\frac{T-70}{Z_{T}}\cdot \frac{RH-80}{Z_{RH}}\right)}\cdot dt$
*D*_ref_ *	0.60 min
*Z*_T_	12.62 °C
*Z*_RH_	22.60%
RMSE	0.92
R^2^	0.86

* The reference temperature and RH are 70 °C and 80%.

**Table 3:** *D*_ref_ (70 °C, 80% RH) values of *Salmonella* spp. in different low-moisture matrices.

Condition	*D*-Value	Estimated *D*_ref_ (min) *	Reference
Sand under controlled RH	$logD_{80℃}=-3.2314\cdot RH+2.7481$ (min)	6.9	[3]
Low-moisture foods	$D_{80℃}=652.37e^{-7.241\cdot RH}$ (min)	4.5	[29]
White grape juice concentrate	$0.5\;min<D_{62℃}<0.8\;min$	0.9–1.4	[37]
Diced apple cubes	-	0.60	This work
Diced apple cubes in hot ascorbic acid	$D_{65℃}=12.3\;s$	0.63	[33]
Apple and orange juices	$D_{71℃}<1\;s$	<0.16	[38]

* Estimated *D*_ref_ values (at 70 °C, 80% RH) were calculated based on the *Z*_T_ and *Z*_RH_ values listed in Table 2.

**Table 4:** *Z*_T_ values of *Salmonella* spp. in low-moisture foods at specific ranges of application.

Food Matrix	Range of Application		*Z*_T_ (°C)	Reference
	T (°C)	RH (%)		
Milk chocolate	70–80	33	18.8 ± 2.5	[39,40]
	70–80	44	20.6 ± 4.1	
	70–80	52	18.1 ± 0.5	
Peanut butter	70–100	33	15.4	[30]
	70–100	53	12.6	
Diced apple cubes	30–80	20–80	12.6	This work
Soy protein powder	80–99	25–32	12.5	[41]
	80–99	36–43	13.2	
	80–99	47–52	13.1	
	75–95	52–58	11.6	
	70–90	58–62	11.2	
	70–85	68–70	10.8	
	70–85	74–76	8.0	
	60–75	84–86	6.7	

**Table 5:** *Z*_T_ values of *Salmonella* spp. in low-moisture foods at specific ranges of application.

Food Matrix	Range of Application		*Z*_RH_ (%)	Reference
	T (°C)	RH (%)		
Soy protein powder	70	58–74	44	[41]
	75	52–75	39	
	80	25–69	41	
Wheat flour	80	31–78	32.2	[29]
Sand (SiO_2_)	80	18–72	31	[3]
Whey protein	80	32–85	30.9	[29]
Almond flour	80	32–81	28.9	[29]
Diced apple cubes	30–80	20–80	22.6	This work
Black peppercorn	80	60–80	21.3	[25]

## Data Availability

The original contributions presented in this study are included in the article. Further inquiries can be directed to the corresponding author.
